# Roles of Parenting Practices on Risky Sexual Behaviour among Adolescents in Rural Cambodia

**Published:** 2019-12

**Authors:** Jihyon PAHN, Youngran YANG

**Affiliations:** 1. Graduate School of Nursing, Jeonbuk National University, Jeonju, Republic of Korea; 2. School of Nursing, Research Institute of Nursing Science, Jeonbuk National University, Jeonju, Republic of Korea

## Dear Editor-in-Chief

Adolescents are classified as high risk for HIV infection worldwide (1). After 2000, worldwide AIDS-related mortality rates have declined over other ages, but it has tripled among those aged 10 to 19 (2).

Parents traditionally have a primary influence on adolescents' sexual behaviour and serve as a protective factor for their children’s sexual behaviour. Reports claim that supportive and repetitive parent-adolescent conversations about sexuality lead to a reduction in frequency of sexual intercourse and number of sex partners among those adolescents (3). High parental monitoring reduced the likelihood of risky sexual practices among young people, for example, adolescent perceiving less parental monitoring were more likely to not using a condom, and having multiple and risky sex partners (4). Cambodia is a country reporting high sex experience and HIV prevalence among adolescent and has one of the highest incidence of HIV in Southeast Asia with a 50% of new HIV infection occurrence among youth aged 15 to 24. However, there is no research on the effects of parenting practices such as parent-adolescent communication and parental monitoring on risky sexual behaviours among rural adolescents in Cambodia.

The purpose of this study was to examine the association of parental monitoring and parental communication with risky sexual behaviour among secondary school students in rural Cambodia.

We conducted this cross-sectional study among 503 students from 3^rd^ year of high schools recruited from 3 rural provinces. Parent-adolescent communication was measured by a total of five items (5). It covers conversation topics of contraction, HIV/AIDS, HIV prevention, condoms, and premarital intercourse. The Parental Monitoring Scale (PMS-8) developed by Small and Kerns (6). It is an 8 item scale assigned to three domains, parental knowledge, parental control, youth disclosure, that assess the youth’s perception about whether their parents are aware of their activities and parental monitoring activities. Based on the literature review, knowledge of HIV/ADIS (9 items) and attitude of HIV/AIDS (4 items), general characteristics such as drinking, smoking and type of cohabitant were measured as factors influencing risk-taking sexual behaviour of adolescents. Risky sexual behaviour was measured using 4 items developed by Le & Kato (7): (a) ‘Have you ever had sex before?’, (b) ‘How old were you the first time you had sex?’, (c) ‘How many partners have you had sex with?’, and (d) ‘Did you use protection (e.g., condom) when you had sex?’. Self-administered questionnaire was used to collect data from July to August 2017. The study was approved by Jeonbuk National University (IRB NO.2017-06-013-001) and the Ministry of Education, Youth and Sport, Cambodia.

Multivariate regression analysis was conducted to assess the predictors of risky sexual behaviour using the SPSS Win 23 program. In regression analysis, knowledge and attitudes of HIV/AIDS, parent-adolescent communication, parental monitoring were entered (Model 1), and gender, drinking status, and cohabitation type, which showed significant difference to scores of the risky sexual behaviour, were added (Model 2).

The average age for the total participants was 18.26 yr; 42.1% were male and 57.9% were female. Participants reported low levels of parent-adolescent communication on sexual matters (11.69 ± 4.58, out of 0–35) and parental monitoring (21.52±6.92, out of 8–40). Among 503 participants, 14 (2.78%) of the students reported that they had sexual experience(s). Among theose 14, 85.7% had their first sexual encounter at age 18 or older. Table 1 shows the related factors of risky sexual behaviour. Higher HIV/AIDS knowledge was associated with higher risky sexual behaviour (*β* = .098, *P* = .037) and parental monitoring (*β* = −.101, *P* =.028) (Model 1). Parent-adolescent communication was not significant. In Model 2, drinking (*β* = .250, *P* < .001), and living with step-parent (*β* = .120, *P = .*005) were associated with higher risky sexual behaviour but the significance of HIV/AIDS knowledge and parental monitoring did not remain. Being male, drinking, having higher HIV/AIDS knowledge level and residing with step-parents were associated with more risky sexual behaviour.

**Table 1:** Relationship of parental practices and sexual risky behaviour of adolescents (N=503)

***Variable***	***Model 1***			***Model 2***						
	**β**	**t**	**p**	**β**	**t**	**p**
HIV/AIDS knowledge	.098	2.092	.037	.056	1.188	.235
Attitude toward HIV/ADIS	−.032	−.699	.485	−.013	−.281	.779
Parent-adolescent communication	.014	.303	.762	.007	.153	.878
Parental monitoring	−.101	−2.202	.028	−.054	−1.189	.235
Sex (Male)^a^				.019	.382	.703
Drinking (Yes)^b^				.250	5.384	<.001
Single-parent^c^				.032	.722	.471
Step-parent^c^				.120	2.793	.005
Relatives^c^				−.002	−.051	.960
R^2^		.011			.082	
F		2.451			5.972	
P		.045			<.001	
ΔR^2^		0.019			.079	

Note.

a female as reference,

b no drinking as reference,

c two-parent as reference

A high level of parental monitoring among adolescents of this study was associated with less involvement in risky sexual behaviour. Parental involvement in prevention programs by monitoring their children’s activities may help reduce adolescents’ affiliation with sexually risky behaviours. The results can provide fundamental information for the development of sexual education and HIV prevention programs for adolescents.
